# Do men and women define health differently? A cross-national study of gender differences in self-rated health

**DOI:** 10.3389/fpubh.2026.1753078

**Published:** 2026-04-21

**Authors:** Henriette Engelhardt, Liliya Leopold

**Affiliations:** 1 Institute for Sociology, University of Bamberg, Bamberg, Germany; 2 Department of Sociology, Universiteit van Amsterdam, Amsterdam, Netherlands

**Keywords:** self-rated health, gender, validity, panel data, international comparison

## Abstract

**Introduction:**

Self-rated health (SRH) is a common measure for examining gender differences in health. However, it is unclear if men and women assess similar health factors in their ratings, particularly outside the US. This study expands on previous research in Germany, Italy, and Sweden–countries with varying gender norms.

**Methods:**

We analyze how chronic conditions, mental health, physical functioning, and health behaviors affect SRH ratings among 50,912 respondents aged 50–79, using panel data from SHARE and HRS and random effects multinomial logistic models.

**Results:**

Our findings indicate that women in the US and Italy are less likely than men to report very good health, while no gender differences were found in Germany and Sweden. Additionally, women in all countries except Germany were more likely to report poor health. Despite variations in gender gaps in SRH across the four countries, the underlying meaning of SRH did not differ between men and women, as health indicators contributed comparably to their ratings. The only exception was mental health, where women reported better SRH than men despite similar levels of depression.

**Conclusion:**

Overall, our results suggest that SRH is a comparable measure for assessing gender differences in health across these countries.

## 1 Introduction

Self-rated health (SRH) is a widely used indicator of population health (39). It is easily collected through surveys via a single question with five response options, ranging from “excellent” to “very poor” (1). Despite its simplicity, SRH has proven to be highly reliable (2), strongly correlated with objective health measures (3), and a robust predictor of morbidity and mortality (4–6). These qualities make SRH a valuable tool in epidemiological and public health research, offering important insights into health disparities across populations.

Despite its benefits, the applicability of SRH for group comparisons has been questioned, as similar levels of SRH may reflect very different actual health problems and levels of physical and mental health in different groups (7–10). Individuals from different societal groups may use information about diagnoses, prescribed medications, and other information differently in their SRH responses (11). Moreover, responses may vary due to cultural differences, previous experiences and the interpretation of the question and response categories (12). Differences in the meaning of SRH have been also proposed as a substantive explanation for the SRH-gender gap. For instance, studies suggest that differences in the interpretation of the SRH instrument between women and men may partly explain the commonly observed “gender health paradox,” where women report worse SRH levels than men despite having longer life expectancy (8, 13–16). For instance, studies suggest that women may have greater somatic awareness and are more likely to acknowledge health problems and seek medical advice as compared to men (10). Consequently, women's responses to the SRH question may more strongly reflect diagnosed chronic conditions. Another common argument is that women may place greater emphasis on mental health issues compared to men, particularly among older generations (8). This could be partly attributed to traditional gender norms, where acknowledging mental health problems may be perceived as a sign of weakness for men who adhere to such roles (17). Although empirical evidence supporting these arguments is somewhat mixed (18, 19), studies suggest that despite potential differences in how SRH is interpreted by women and men, SRH generally captures similar health dimensions across genders (8, 26).

While most of previous studies are based on U.S. data, research suggests that gender differences in the underlying meaning of SRH may also vary across countries (9, 20–23). Studies consistently find variations in size and direction of gender gap in SRH between countries (14, 24, 25) and argue that this might be due to differences in gender norms and related levels of gender equality in acess to resources (15, 24). Accordingly, in more egalitarian societies, the gender gap in health may be smaller as compared to less egalitarian countries with more traditional gender norms. In turn, country differences in gender norms might be related to differences in meaning of SRH beween women and men. In more egalitarian countries with less traditional gender norms, SRH meaning might be more consistent for both genders. Conversely, in more traditional societies, where women are more often tied to caregiving roles than men and men are more tied to breadwinner roles than women, the meaning of SRH may diverge more significantly between genders. In these contexts, women may place more weight on mental, physical and caregiving-related stressors in their assessment of health, while men may be less sensitive to their physical health problems or disregard mental or emotional issues due to traditional gender norms that discourage expressing vulnerability in men. Despite these ideas have been articulated in previous research (8, 26), evidence on contextual differences in meaning of SRH between women and men remains underexplored in existing research.

### 1.1 The present study

The present study aims at expanding the comparative scope of previous research. We provide a cross-country comparison of gender differences in SRH by analyzing data from the U.S. – the context most commongly studied so far with three European contexts: Germany, Italy, and Sweden. These four countries differ in terms of gender equity and gender norms, whereby Italy represents a highly traditional context with large differences in gender roles between women and men, Sweden a highly egalitarian context with smallest differences in gender roles; and Germany and the United States can be placed in between. In this study we will empirically assess whether these differences translate into gender differences in meaning of SRH between countries. Following previous research from the U.S., we assess whether the structure of SRH with respect to its associations with chronic conditions, functional limitations, mental health, and health behaviors varies between women and men in the four countries using panel data from the Health and Retirement Study and the Survey of Health, Aging and the Survey of Health, Aging and Retirement from 2004 to 2020.

## 2 Data and methods

### 2.1 Data

Data were from two large aging studies, the Survey of Health, Aging and Retirement in Europe (SHARE) and the Health and Retirement Study (HRS). SHARE and HRS are sister-studies with data collection and measures harmonized to enable cross-national comparisons. Begun in 1992, HRS is a household sample including approximately 20,000 people (27, 28). The interviews are collected using a combination of in-person and telephone interviews ^1^. The first wave of the SHARE survey was collected in 2004 (29). Sampling designs vary across SHARE countries ^2^.

For the sake of comparison, our analytical sample covers data up to 16 years from 2004 to 2020 (HRS, wave 7 to wave 15) respectively 2019 (SHARE, wave 1 to wave 8) with respondents aged 50–79 who answered the self-rated health questions. With these selections, we can draw on 29,165 persons (137,734 person-years) from the U.S., 8,006 persons (21,257 person-years) from Germany, 7,796 persons (22,411 person-years) from Italy and 5,945 persons (18,308 person-years) from Sweden for the statistical analyses. Table 1 shows descriptive statistics for all measures by gender and country.

**Table 1:** Selected characteristics of the analytic sample, by gender and country.

Measure	U.S.			Germany			Italy			Sweden		
	M	F	Δ^b^	M	F	Δ^b^	M	F	Δ^b^	M	F	Δ^b^
SRH												
Very good (0/1)	0.394	0.387	^**^	0.209	0.206		0.242	0.212	^***^	0.438	0.426	
Good (0/1)	0.329	0.32	^***^	0.406	0.42	^*^	0.399	0.362	^***^	0.353	0.333	^**^
Poor (0/1)	0.278	0.293	^***^	0.386	0.374		0.359	0.426	^***^	0.209	0.24	^***^
Chronic conditions												
Heart disease (0/1)	0.264	0.221	^***^	0.138	0.067	^***^	0.113	0.063	^***^	0.141	0.075	^***^
Hypertension (0/1)	0.622	0.616		0.432	0.398	^***^	0.411	0.393	^*^	0.37	0.352	^*^
Stroke (0/1)	0.082	0.07	^***^	0.046	0.029	^***^	0.032	0.02	^***^	0.044	0.027	^***^
Diabetes (0/1)	0.277	0.258	^***^	0.153	0.107	^***^	0.131	0.1	^***^	0.126	0.079	^***^
Lung disease (0/1)	0.104	0.136	^***^	0.067	0.075		0.062	0.044	^***^	0.039	0.043	
Cancer (0/1)	0.138	0.142		0.069	0.069		0.032	0.043	^***^	0.06	0.056	
Functional limitations												
ADL (0–5)	0.27	0.331	^***^	0.166	0.151		0.137	0.177	^***^	0.12	0.109	
IADL (0–5)	0.219	0.27	^***^	0.108	0.093		0.122	0.145	^**^	0.08	0.071	
15.6-8.2,-14.3690ptMobility (US: 0–5; EU: 0–4)	0.858	1.214	^***^	0.306	0.383	^***^	0.42	0.616	^***^	0.188	0.286	^***^
Mental health												
Depression (0/1)^a^	0.177	0.25	^***^	0.157	0.269	^***^	0.221	0.396	^***^	0.113	0.221	^***^
Health behavior												
Smoker (0/1)	0.17	0.016	^***^	0.19	0.128	^***^	0.182	0.114	^***^	0.107	0.14	^***^
Underweight (0/1)	0.007	0.016	^***^	0.002	0.012	^***^	0.002	0.018	^***^	0.004	0.015	^***^
Normalweight (0/1)	0.212	0.271	^***^	0.283	0.407	^***^	0.308	0.435	^***^	0.362	0.452	^***^
Overweight (0/1)	0.434	0.312	^***^	0.487	0.347	^***^	0.521	0.357	^***^	0.462	0.349	^***^
Obese (0/1)	0.34	0.379	^***^	0.22	0.219		0.157	0.174	^**^	0.163	0.158	
Age (years)	64.5	64.3	^***^	65.	64.0	^***^	65.9	64.8	^***^	67.0	66.3	^***^
Persons	12,879	16,286		3,817	4,189		3,588	4,208		2,773	3,172	
Person-years	58,629	79,105		10,077	11,180		10,131	12,280		8,418	9,890	

^a^Measures for depression differ for the US and Europe (US: eight-item CES-D scale, cutoff point ≥ 3; European countries: 12-item Euro-D scale, cutoff point ≥ 4).

^b^t-tests for mean differences of males and females.

p-value for Ha: diff ≠ 0: ^***^p < 0.001, ^**^p < 0.01; ^*^p < 0.05.

### 2.2 Measurement

*Self-rated health* was measured using the question “Would you say your health is...?” with response options, i.e., excellent (1), very good (2), good (3), fair (4), poor (5). To facilitate the interpretation of the results we combind the response options excellent and very good health (hereafter very good health) and the response options fair and poor (hereafter poor health).

*Chronic conditions* were reported in response to the question “Has a doctor ever told you that you had any of the following conditions?” *Heart disease, hypertension, stroke, diabetes, lung disease, cancer*, and *arthritis* were collected in SHARE and HRS and used as dummy variables for each condition.

*Depressive symptoms* were measured in SHARE by the 12-item *Euro-D* scale and in HRS by an eight-item *CES-D* scale (30). The scales used different reference periods: the Euro-D asked about depressive symptoms in the past month, whereas the CES-D asked about symptoms in the past week. In our main analysis, we followed Crimmins et al. (14) and dichotomized the scales at EUR0-D ≥ 4 and CES-D ≥ 3. Additionally, we calculated mean values based on the original scales.

*Health behaviors* included smoking and body mass index (BMI). Participants' *smoking status* was recorded using a binary variable (1 = current smoker, 0 = non-smoker). *BMI* was calculated from self-reported height and weight, and categorized as underweight (BMI < 18.5), normal weight (18.5 ≤ BMI < 25), overweight (25 ≤ BMI < 30), and obese (BMI ≥ 30).

*Functional limitations* were captured in both surveys with five different aspects. For the self-reports, we used count variables for the number of restrictions in activities of daily living and mobility. The *activities of daily living index* (ADL) was measured as a summary index of restrictions in eating, dressing, walking across a room, and getting in or out of bed. The *instrumental activities of daily living index* (IADL) was the sum of having difficulties using a phone, taking medication, handling money, shopping, and preparing a meal. Both ADL and IADL ranged from 0 to 5, with higher values indicating more difficulties. The mobility index was the sum of difficulties walking 100 meters, walking across a room, climbing several flights of stairs, and climbing one flight of stairs. It ranged from 0 to 4, with higher values indicating lower mobility. In HRS, the index additionally included walking several blocks and ranged from 0–5.

Finally, in the empirical analyses, we additionally controlled for selected respondent attributes associated with SRH: *nativity status* (native, foreign-born), *ethno-race* in the U.S. sample, and *educational attainment* (below secondary, secondary, above secondary education).

### 2.3 Analytic strategy

To detect whether and how the underlying structure of SRH may have varied by gender, age, and country, we analyzed whether similar answers on SRH were associated with similar health conditions, and whether even similar conditions contributed differently to ratings of SRH by gender and country. Therefore, we first analyzed the gender-specific distribution of SRH at different ages for the four countries descriptively.

Secondly, to test whether men and women in these countries differ in how health conditions predict their SRH at different ages, we estimated country-specific random effects multinomial logistic models (REMLM) (23, 31). While SRH is inherently ordinal, we treat it as nominal to allow for the possibility that the predictors of health ratings function differently at different ends of the health spectrum. This approach is chosen not only to bypass the proportional odds assumption but to capture potential non-linear effects where health conditions, such as mental health, may exert a stronger or weaker influence on the probability of reporting “Poor” vs. “Good” health compared to “Excellent” vs. “Good” health. The primary inferential target of our REMLM is the log-odds of being in a specific SRH category relative to the “Good” health reference group, conditional on individual random effects. To investigate gender-specific effects we follow Zajacova et al. (8) by including interaction effects of self-reported health measures and gender.

Thirdly, the subjective nature of SRH warranted careful consideration of important contributing factors, particularly mental health (8). As mental health's impact on SRH could have varied across the spectrum of SRH-responses (12), we compared the gender- and age-specific means of CES-D and EURO-D at each level of SRH separately while controlling for physical conditions. This approach allowed us to identify potential non-linear effects. This approach addressed the limitation of previous analyses of SRH (e.g., 8; 9) by allowing for varying effects of mental health at different levels of SRH. All the analyses were performed using STATA version 18.0.

## 3 Results

### 3.1 Gender SRH gaps by country

Table 1 reports the detailed distributions of all characteristics in the country- and gender-specific samples as well as the results on *t*-tests for mean differences between women and men. While women are less likely to report very good health in the U.S. and in Italy than men, there is no SRH gender gap in Germany and Sweden. Furthermore, women in all countries except Germany are more likely to report poor health. In contrast, German women are more likely than men to report good health.

**Figure 1:**
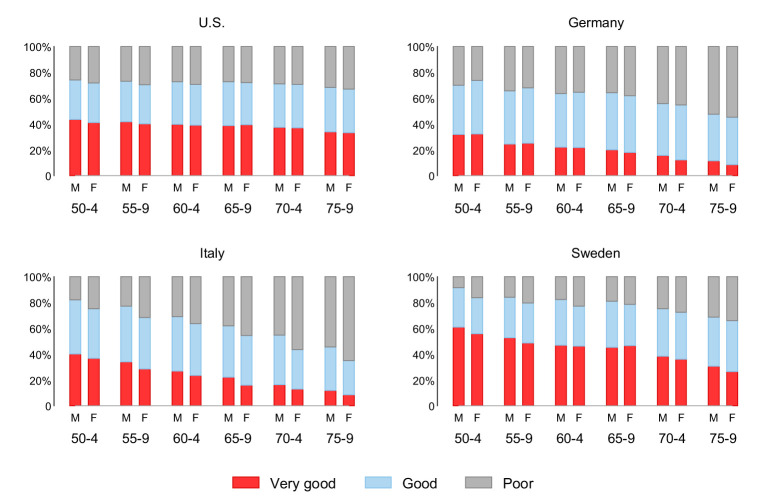
Age-specific distribution of SRH for women and men. Unweighted distributions.

Figure 1 further visually investigates gender gaps in SRH by age. We see a deterioration in SRH with age in all four countries, although the baseline levels and decreases differ between countries. While the baseline level of SRH in Sweden is higher than in the United States, SRH deteriorates stronger with increasing age. The curve is also much flatter in the US than in Germany and Italy. Notably, the proportion of men and women in Germany and Italy who rate their health as poor rather than good is increasing.

### 3.2 Gender differences in effects of health indicators to SRH ratings by country

**Figure 2:**
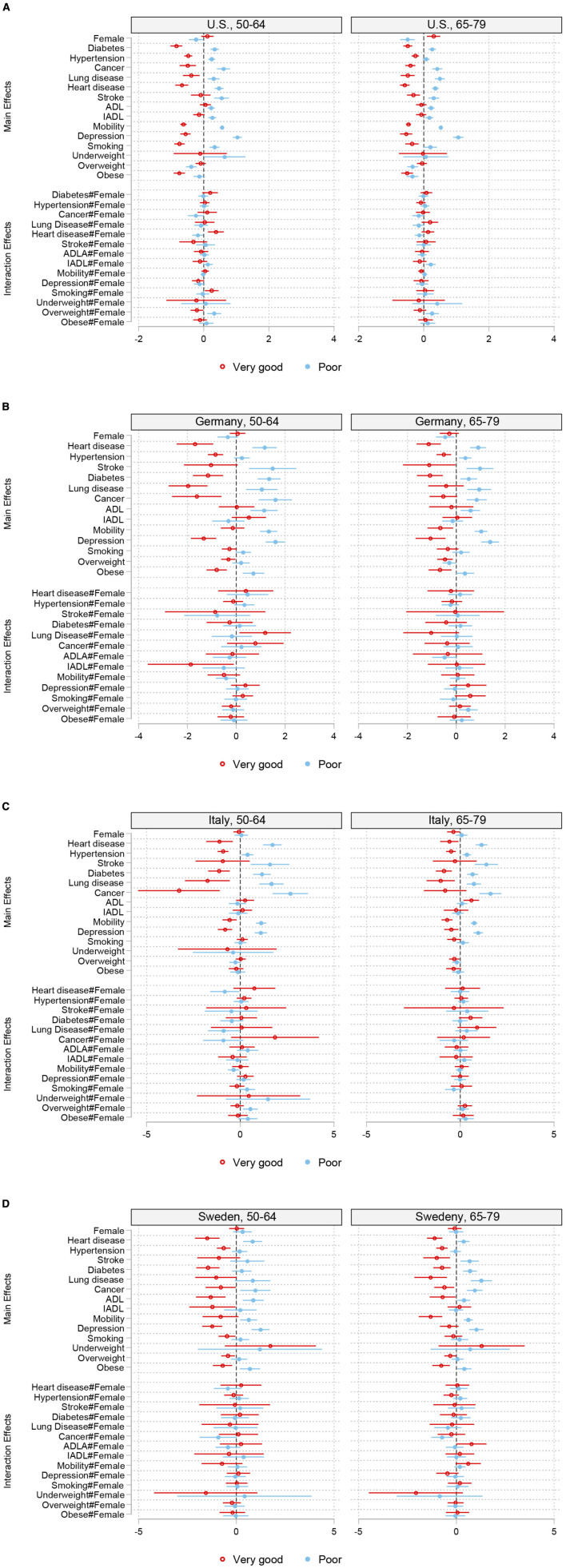
**(A)** Random-effects multinomial logistic models of SRH for women and men by age in the U.S. (95% confidence intervals). Additional control variables not displayed are age, nativity, ethnicity/race, and education. **(B)** Random-effects multinomial logistic models of SRH for women and men by age in Germany (95% confidence intervals). Additional control variables not displayed are age, nativity, and education. Underweight is dropped from the models due to small number of cases. **(C)** Random-effects multinomial logistic models of SRH for women and men by age in Italy (95% confidence intervals). Additional control variables not displayed age, nativity, and education. Underweight is dropped from the model for the age group 65–79 due to small number of cases. **(D)** Random-effects multinomial logistic models of SRH for women and men by age in Sweden (95% confidence intervals). Additional control variables not displayed are age, nativity, and education.

Concerning our main interest, whether men and women differ in how health measures predict their SRH at different ages, we estimated random-effects multinomial logistic models of SRH. Figure 2 show the estimated coefficients of main effects of health indicators as well as their interactions with gender separately for persons having excellent or very good health and fair or poor health in comparison to good health (reference category). The full models are provided in the Appendix Tables A1a–d.

The interpretation of the coefficients is the same as in a cross-sectional multinomial logit model, except that, in the random-effects case, they are to be interpreted as conditional on the random effects. They can be thought of as the natural log of a double ratio: the log of the relative risk, relative to the base category. For instance, the female coefficient for the United States in the age group 65–79, in the first equation (“very good health”) is around 0.3 (Appendix Table A1a). Thus, women are more likely to report very good health than men, relative to reporting good health. Here we focus on the sign of the coefficient that can be meaningfully interpreted.

There are three central patterns that emerge from these analyses. First, statistically significant gender differences in SRH can be found in the older age goup in the U.S. when controlling for differences in health conditions and socioeconomic characteristics. Here, women report poor health significantly less often than men and very good health more often than men. However, for Americans aged 50–64, there are only gender differences in reporting poor health but not in reporting very good health. In the case of Germany and Italy, significant effects are found only for selected outcomes in the 65+ age group. In Sweden, there are no gender differences in SRH at any age, controlling for other health indicators.

Second, both age groups are similar in the association of health measures and SRH. Third, most of our health indicators have opposite effects on the likelihood of reporting very good health and the likelihood of reporting poor health (relative to good health). For example, mobility problems increase (decrease) the likelihood of reporting poor (very good) health. However, the strength of the effects on the two opposite categories is not symmetrical. In the US, for example, the negative effect of diabetes on very good health is much stronger than the positive effect on poor health in the 50–64 age group. As for the interaction terms, individual asymmetric effects are significant, e.g. cancer, heart disease and obesity in the younger American age group.

### 3.3 Gender differences in meaning of SRH by country

**Figure 3:**
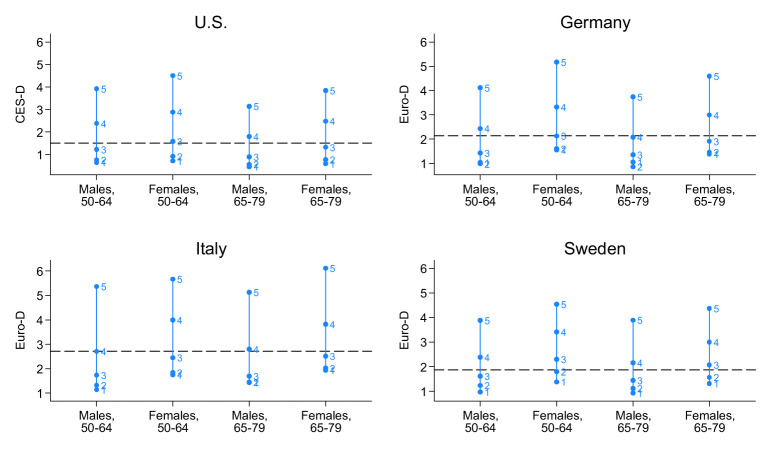
Mean values of depression scale CES-D and Euro-D according to SRH for women and men by age and country. Levels of SRH: excellent (1), very good (2), good (3), fair (4), poor (5). US: eight-item CES-D scale; European countries: 12-item Euro-D scale.

We proceed to examine the self-rated health (SRH) responses in relation to mental health status. Figure 3 illustrates the gender-specific average scores for the CES-D (ranging from 0 to 8) and Euro-D (ranging from 0 to 12) across the original five SRH categories, classified by age group. The numerical data, along with 95% confidence intervals, can be found in Appendix Table A1.

Our findings indicate that although poorer SRH is consistently linked to a higher prevalence of mental health issues, men and women with comparable mental health conditions tend to perceive their health differently. Specifically, across all four countries, women exhibit a more optimistic view of their health than men relative to their mean depression level. For example, in Germany, men aged 65+ with an average Euro-D score of 2.1 typically rate their SRH as “fair,” whereas women of the same age and with the same depression score rate their health more positively as “good”.This bivariate result is also confirmed under control of physical conditions in an estimation of mean mental health at each level of SRH using linear RE-models (see Appendix Table A2). Reporting styles differ in all countries significantly by gender, as can be seen from the overlapping confidence intervals.

Moreover, in the US and in Sweden the confidence intervals of mean depression for each SRH category do not overlap for both men and women of all ages even under control of physical conditions. Thus, the SRH categories discriminate well between different mental states. However, in Germany and Italy women and men do not distinguish between “excellent” and “very good” with different levels of mental health as confidence intervals of mean depression overlap.

### 3.4 Robustness checks

We also conducted additional analysis to explore the robustness of our findings. First, we replicated all multivariate analysis without controls. The results are substantially identical to those reported above (available from the authors upon request).

Second, to account for possible cohort effects (32), we estimated random-effects ordered logit models on self-rated health including age and cohort (Appendix Figure A1). No cohort effects signifcantly different from zero were found in Germany. However, the estimated effects suggest an increasing trend toward worse SRH in both the United States and Sweden, and a decreasing trend toward better SRH in Italy. In none of the four countries, however, are there any systematic gender differences over time.

## 4 Summary

Using internationally comparable panel data from SHARE and the HRS, we investigated whether potential gender differences in meaning of SRH vary between countries. To test whether men and women in the US, Germany, Italy, and Sweden differed in how health conditions predicted their SRH at different ages, we estimated country-specific random effects multinomial logistic models with gender-specific interaction effects.

Our findings reveal country-specific differences in the gender-specific distribution of SRH, with women in Italy and the US reporting very good health less often than men. No gender differences emerged in the upper distribution of SRH in Germany and Sweden. Furthermore, women in all countries except Germany were more likely to report poor health. In Germany, significant gender differences only exist in the middle range of SRH, with women more often reporting good health. Thus, average country-specific differences in SRH between men and women mask gender-specific differences in assessment patterns.

Regardless of these country-specific differences, our study shows that women and men are remarkably similar in how they incorporate a wide range of chronic health conditions, functional limitations, mental health, and health behaviors in their SRH evaluations, and this was largely consistent across countries. This gender equality in the structure of SRH supports findings from the US for men and women aged 25–84, where the associations between health indicators and SRH were very similar between men and women (40). Our findings are also consistent with Case and Paxson's (13) study using NHIS data, where chronic conditions predicted SRH similarly for men and women, and with findings from Spain, where SRH ratings were similar for men and women after adjusting for differences in health conditions (33).

However, the gender comparison across age is sensitive to the inclusion of covariates. Taking health measures into account, women in the U.S. and in Germany are less likely to report poor health than men and do increasingly so in the older age group. For the United States, based on ordered logit models Zajacova et al. (8) finds that men experience a steeper worsening in SRH across age than women do. Our results, which are based on a different model, can also be interpreted cautiously as a greater deterioration in self-reported health among American and German men at older ages.

As far as possible age effects in the structure of SRH are concerned, our analysis does not give us a clear picture. Based on our results, it appears that the effects of some of the health indicators decrease with increasing age, both for men and for women. However, further analysis is needed to draw such conclusions, as our models do not allow a direct comparison of the estimated coefficients.

Another finding of our study was that SRH categories differed only slightly between men and women in terms of mental health. With the same research question, Zajacova et al. (8) conducted a conceptual Bayesian analysis. Irrespective of the differences in methodological approaches, our main results are consistent: at worse levels of SRH, the distribution of mental health become better separated. The gender-specific difference is modest relative to the pattern across SRH levels. This means that men and women do not differ systematically in their assessment of the different categories of subjective health. This result holds even when the underlying physical conditions are taken into account.

The observed cross-country variations in self-rated health trends and gender gaps reflect distinct sociocultural and institutional landscapes. While we identified an increasing trend toward worse SRH in both the United States and Sweden, Italy demonstrated a particularly notable decrease in the likelihood of reporting good health alongside a widening gender gap. These patterns may be driven by Italy's status as a “highly traditional” context, where rigid gender roles often tie women to intensive caregiving responsibilities while men are primarily viewed as breadwinners. Such traditional norms can lead to disparate levels of exposure to chronic stressors and unequal access to socioeconomic resources, potentially exacerbating the gender health gap in older age. In contrast, the smaller gender gap in Sweden aligns with its highly egalitarian norms and institutional support systems, which facilitate more equitable access to health-promoting resources. Furthermore, the general downward trends in health assessments in the U.S. and Europe may stem from shifting physical and mental health distributions—such as the increasing proportion of individuals reporting poor health in Italy and Germany—rather than differences in how health is subjectively defined. Our finding that the meaning of SRH remains consistent across these diverse contexts suggests that the diverging trends are likely to be rooted in genuine differences in health status and social structure, rather than variations in cultural reporting styles. Exploring the gender health paradox (10) in diverse settings beyond the US is essential to fully understand these complex patterns.

Furthermore, as Zajacova et al. (8) and our analysis has shown, a promising step forward could be to extend the analyses beyond a focus on central tendencies and away from analyzing SRH as a continuous or dichotomous variable, as is mostly done, and to explicitly consider the individual categories. While the original data in our study used a five-point scale, we collapsed these into “Very Good,” “Good,” and “Poor” primarily to ensure statistical stability in our complex random-effects multinomial models, particularly given the low cell counts in extreme categories when stratified by country and age. This approach also enhances cross-national comparability by reducing the influence of country-specific reporting styles at the scale's extremes. We acknowledge that this results in some information loss, specifically the distinction between peak health (“Excellent”) and high health (“Very Good”), as well as the nuance between moderate (“Fair”) and severe (“Poor”) health limitations. However, our bivariate analyses using the original five categories (see Figure 3 and Appendix Table A1) confirm that the associations between mental health indicators and SRH remain consistent in direction across all levels, suggesting that the primary conclusions of our multivariate models remain robust despite the reduction in categories.

To ensure cross-national comparability, we used validated clinical cut-points for depressive symptoms: for the EURO-D (12 items) and for the CES-D (eight items), following Crimmins et al. While these instruments utilize different reference periods-−1 month and 1 week, respectively—both are standardized tools for identifying clinically significant symptoms in their respective survey contexts. Similarly, our functional indices were harmonized to be as conceptually equivalent as possible. Because our primary objective is to analyze the internal association between health indicators and SRH within each country—rather than comparing absolute prevalence levels—these minor measurement variations do not undermine the validity of our cross-national conclusions.

## 5 Limitations

First, most health measures in our analyses were self-reported which means that possible influences on the SRH judgment may also influence the reporting of health measures. This was because data on objectively measured health markers were limited in data at hand: data on biological risk markers were only available in HRS; the data from dried blood spots in SHARE collected in wave 6 have not been released yet [March 2024; (34)]. Second, related to the previous point health measures used are only a subset of those that matter for health judgement. In particular, there is a lack of information on the severity or duration of health problems, which may distort the results if there are significant differences between men and women.

Third, part of the measures used in the analyses are diagnosed conditions by medical doctors. As men and women differ in medical-care-seeking behavior as well as in diagnoses of medical conditions, these measures could be biased (8). For example, if men are systematically less likely to visit a doctor than women with the same health status, the number of diagnosed and reported conditions would be expected to be lower. More non-self-reported data are therefore needed for a comprehensive international comparison of gender differences in SRH.

Fourthly, the random-effects multinomial logistic models used to analyse the underlying structure of self-rated health are based on the standard assumption of exogeneity, which assumes that unobserved, individual-specific heterogeneity is uncorrelated with observed health indicators. If unobserved factors are correlated with both the health predictors and the subjective health ratings, the estimated coefficients may be biased. Future research could utilize correlated random-effects models, which allow the exogeneity assumption to be relaxed by explicitly accounting for the correlation between individual-specific errors and the independent variables. This would provide further nuance to the longitudinal dynamics of health reporting.

Although our results indicate a largely consistent internal structure of SRH across genders and countries, potential threshold shifts were not explicitly identified or accounted for. Our findings should therefore be interpreted as evidence regarding the differential weighting and associations of health indicators in the subjective evaluation process, rather than as a definitive claim of absolute threshold comparability. As we did not use anchoring vignettes [e.g. (35)] or specific statistical techniques [e.g. (20, 36)] to distinguish between reporting styles and actual health differences, it is possible that men and women use different internal benchmarks for categories such as “good” or “fair” health. Nevertheless, the stability of the predictive weights assigned to various health markers suggests a shared understanding of the relative importance of different health dimensions in overall health assessments.

Moreover, we did not disentangle differences in SRH between men and women into gender-specific differences of health conditions and differences in gender-specific reporting styles. To quantify the underlying structures of the country-specific SRH gap between women and men in the decomposition tradition of Oaxaca and Blinder (37, 38), future statistical work may provide a technique that allows these effects to be separated.

Despite these limitations, our research revealed that both men and women in different countres consider a diverse array of health indicators when assessing their health. Furthermore, individuals over the age of 50 evaluate these indicators in a largely similar manner, with the exception of mental health issues. This indicates a shared understanding of the significance of various health dimensions in their overall health evaluations across both genders, thereby reinforcing the robustness of self-reported health measures in international population health studies.

During the preparation of this work the author(s) used Jenni.ai in order to improve language and readability. After using this tool, the author(s) reviewed and edited the content as needed and take(s) full responsibility for the content of the publication.

## Data Availability

Publicly available datasets were analyzed in this study. This data can be found at: https://share-eric.eu.

## Footnotes

1 The Health and Retirement Study data is sponsored by the National Institute on Aging (grant number U01AG009740) and is conducted by the University of Michigan.

2 This paper uses data from SHARE Waves 1, 2, 4, 5, 6, 7, and 8 (DOIs: 10.6103/SHARE.w1.800, 10.6103/SHARE.w2.800, 10.6103/SHARE.w4.800, 10.6103/SHARE.w5.800, 10.6103/SHARE.w6.800, 10.6103/SHARE.w7.800, 10.6103/SHARE.w8.800, 10.6103/SHARE.w8ca.800) see Börsch-Supan et al. (29) for methodological details (1). The SHARE data collection has been funded by the European Commission, DG RTD through FP5 (QLK6-CT-2001-00360), FP6 (SHARE-I3: RII-CT-2006-062193, COMPARE: CIT5-CT-2005-028857, SHARELIFE: CIT4-CT-2006-028812), FP7 (SHARE-PREP: GA N°211909, SHARE-LEAP: GA N°227822, SHARE M4: GA N°261982, DASISH: GA N 283646) and Horizon 2020 (SHARE-DEV3: GA N°676536, SHARE-COHESION: GA N°870628, SERISS: GA N°654221, SSHOC: GA N°823782, SHARE-COVID19: GA N°101015924) and by DG Employment, Social Affairs & Inclusion through VS 2015/0195, VS 2016/0135, VS 2018/0285, VS 2019/0332, and VS 2020/0313. Additional funding from the German Ministry of Education and Research, the Max Planck Society for the Advancement of Science, the U.S. National Institute on Aging (U01_AG09740-13S2, P01_AG005842, P01_AG08291, P30_AG12815, R21_AG025169, Y1-AG-4553-01, IAG_BSR06-11, OGHA_04-064, HHSN271201300071C, RAG052527A) and from various national funding sources is gratefully acknowledged (see www.share-project.org).
