# Supplementary material for: Do men and women define health differently? A cross-national study of gender differences in self-rated health
Source: Front Public Health. 2026 Apr 21;14:1753078. doi: 10.3389/fpubh.2026.1753078 (PMC13138979; doi:10.3389/fpubh.2026.1753078)
Supplement: Supplementary file 1 [file Supplementary_file_1.docx]

**Supplement**

Figure A1: Random-effects ordered logistic models of SRH for women and men with age, and cohort

Note: All models additionally control for nativity, race/ethnicity (U.S. only), and education.

Table A1a: Regression results for Figure 2a, U.S.

|  | 50-64 | | 65-79 | |
| --- | --- | --- | --- | --- |
|  | Very good | Poor | Very good | Poor |
| Female | 0.109 | -0.236^*^ | 0.303^**^ | -0.497^***^ |
|  | (1.22) | (-2.33) | (3.16) | (-4.79) |
| Age | -0.00664 | -0.0311^***^ | -0.0258^***^ | -0.000868 |
|  | (-1.68) | (-7.14) | (-6.64) | (-0.20) |
| Native | 0.530^***^ | -0.631^***^ | 0.572^***^ | -0.799^***^ |
|  | (8.35) | (-10.35) | (7.49) | (-11.08) |
| Below secondary educ. | -0.461^***^ | 1.195^***^ | -0.326^***^ | 1.096^***^ |
|  | (-6.88) | (20.51) | (-5.32) | (19.22) |
| Above secondary educ. | 0.796^***^ | -0.648^***^ | 0.628^***^ | -0.560^***^ |
|  | (16.59) | (-11.15) | (11.83) | (-8.88) |
| Black | -0.485^***^ | 0.181^***^ | -0.670^***^ | 0.261^***^ |
|  | (-9.64) | (3.63) | (-10.78) | (4.30) |
| Other | -0.485^***^ | 0.357^***^ | -0.483^***^ | 0.424^***^ |
|  | (-6.94) | (5.53) | (-4.66) | (4.42) |
| Diabetes | -0.842^***^ | 0.333^***^ | -0.490^***^ | 0.259^***^ |
|  | (-9.66) | (5.96) | (-7.83) | (5.10) |
| Hypertension | -0.476^***^ | 0.241^***^ | -0.257^***^ | 0.0726 |
|  | (-8.92) | (5.18) | (-5.14) | (1.58) |
| Cancer | -0.494^***^ | 0.606^***^ | -0.411^***^ | 0.410^***^ |
|  | (-4.15) | (6.40) | (-5.74) | (5.88) |
| Lung disease | -0.385^**^ | 0.305^***^ | -0.492^***^ | 0.492^***^ |
|  | (-3.17) | (3.70) | (-4.80) | (6.93) |
| Heart disease | -0.673^***^ | 0.468^***^ | -0.581^***^ | 0.357^***^ |
|  | (-7.06) | (7.49) | (-8.56) | (7.63) |
| Stroke | -0.0961 | 0.545^***^ | -0.317^***^ | 0.305^***^ |
|  | (-0.66) | (5.10) | (-3.35) | (3.93) |
| ADL | 0.0506 | 0.224^***^ | -0.0786 | 0.222^***^ |
|  | (0.63) | (4.48) | (-1.04) | (4.65) |
| IADL | -0.147 | 0.261^***^ | -0.0810 | 0.169^**^ |
|  | (-1.83) | (4.67) | (-1.10) | (3.10) |
| Mobility | -0.626^***^ | 0.560^***^ | -0.470^***^ | 0.523^***^ |
|  | (-15.49) | (21.04) | (-15.69) | (20.67) |
| Depression | -0.560^***^ | 1.032^***^ | -0.538^***^ | 1.059^***^ |
|  | (-7.86) | (16.51) | (-6.03) | (14.73) |
| Smoking | -0.749^***^ | 0.332^***^ | -0.361^***^ | 0.199^*^ |
|  | (-10.19) | (4.69) | (-3.72) | (2.12) |
| Underweight | -0.109 | 0.639^*^ | -0.0276 | 0.0611 |
|  | (-0.27) | (2.02) | (-0.08) | (0.18) |
| Overweight | -0.0945 | -0.384^***^ | -0.0545 | -0.344^***^ |
|  | (-1.33) | (-5.00) | (-0.78) | (-4.59) |
| Obese | -0.756^***^ | -0.139 | -0.513^***^ | -0.348^***^ |
|  | (-9.35) | (-1.68) | (-6.20) | (-4.14) |
| Diabetes#Female | 0.195 | -0.0137 | 0.0797 | -0.0110 |
|  | (1.70) | (-0.19) | (0.95) | (-0.16) |
| Hypertension#Female | 0.0316 | 0.00800 | -0.0892 | 0.0369 |
|  | (0.46) | (0.13) | (-1.35) | (0.61) |
| Cancer#Female | 0.103 | -0.245^*^ | -0.0253 | -0.157 |
|  | (0.71) | (-2.15) | (-0.25) | (-1.74) |
| Lung Disease#Female | 0.0290 | -0.0850 | 0.190 | -0.157 |
|  | (0.20) | (-0.86) | (1.54) | (-1.81) |
| Heart disease#Female | 0.372^**^ | -0.182^*^ | 0.129 | -0.141^*^ |
|  | (3.22) | (-2.34) | (1.43) | (-2.33) |
| Stroke#Female | -0.321 | 0.0581 | 0.0678 | -0.00754 |
|  | (-1.52) | (0.42) | (0.48) | (-0.07) |
| ADLA#Female | -0.0844 | 0.0155 | -0.0539 | -0.0503 |
|  | (-0.80) | (0.25) | (-0.53) | (-0.83) |
| IADL#Female | -0.118 | 0.125 | -0.124 | 0.213^**^ |
|  | (-1.09) | (1.78) | (-1.23) | (3.10) |
| Mobility#Female | 0.0443 | -0.0173 | -0.0785^*^ | 0.0253 |
|  | (0.93) | (-0.53) | (-2.06) | (0.79) |
| Depression#Female | -0.173 | -0.132 | -0.0816 | -0.0519 |
|  | (-1.93) | (-1.68) | (-0.76) | (-0.59) |
| Smoking#Female | 0.242^*^ | -0.0359 | 0.0380 | 0.0451 |
|  | (2.47) | (-0.38) | (0.29) | (0.36) |
| Underweight#Female | -0.227 | 0.0647 | -0.157 | 0.410 |
|  | (-0.49) | (0.17) | (-0.39) | (1.07) |
| Overweight#Female | -0.211^*^ | 0.318^**^ | -0.120 | 0.252^*^ |
|  | (-2.35) | (3.19) | (-1.32) | (2.55) |
| Obese#Female | -0.114 | 0.0828 | 0.0556 | 0.121 |
|  | (-1.14) | (0.79) | (0.53) | (1.11) |
| Constant | 1.395^***^ | 0.137 | 2.340^***^ | -1.348^***^ |
|  | (5.72) | (0.51) | (7.98) | (-4.19) |
| var(u1) | 3.633^***^ | | 3.689^***^ | |
|  | (32.00) | | (30.97) | |
| var(u2) | 2.969^***^ | | 3.426^***^ | |
|  | (26.51) | | (26.08) | |
| Observations | 68167 | | 61791 | |
| *BIC* | 101971.1 | | 95790.2 | |

Note: t statistics in parentheses; ^*^ p < 0.05, ^**^ p < 0.01, ^***^ p < 0.001.

Table A1b: Regression results for Figure 2b, Germany

|  | 50-64 | | 65-79 | |
| --- | --- | --- | --- | --- |
|  | Very good | Poor | Very good | Poor |
| Female | 0.0502 | -0.350 | -0.288 | -0.457^*^ |
|  | (0.33) | (-1.72) | (-1.56) | (-2.51) |
| Age | -0.0444^***^ | 0.0209 | -0.0587^***^ | 0.0560^***^ |
|  | (-4.79) | (1.95) | (-4.77) | (5.58) |
| Nativ | 0.307^*^ | -0.502^***^ | -0.146 | -0.359^**^ |
|  | (2.20) | (-3.49) | (-1.03) | (-2.96) |
| Below secondary educ. | 0.0452 | 0.792^***^ | -0.274 | 0.215 |
|  | (0.25) | (4.43) | (-1.53) | (1.67) |
| Above secondary educ. | 0.650^***^ | -0.459^***^ | 0.559^***^ | -0.511^***^ |
|  | (6.86) | (-3.98) | (4.89) | (-4.95) |
| Heart disease | -1.693^***^ | 1.159^***^ | -1.133^***^ | 0.892^***^ |
|  | (-4.57) | (4.78) | (-4.64) | (5.67) |
| Hypertension | -0.856^***^ | 0.226 | -0.517^***^ | 0.369^**^ |
|  | (-5.73) | (1.48) | (-3.63) | (3.09) |
| Stroke | -1.042 | 1.485^**^ | -1.110^*^ | 0.966^***^ |
|  | (-1.88) | (3.07) | (-2.06) | (3.60) |
| Diabetes | -1.160^***^ | 1.344^***^ | -1.080^***^ | 0.509^**^ |
|  | (-3.82) | (5.93) | (-4.14) | (3.15) |
| Lung disease | -1.977^***^ | 1.043^***^ | -0.420 | 0.937^***^ |
|  | (-5.01) | (3.29) | (-1.17) | (3.94) |
| Cancer | -1.620^**^ | 1.598^***^ | -0.534 | 0.835^***^ |
|  | (-3.20) | (4.74) | (-1.93) | (4.25) |
| ADL | 0.0138 | 1.137^***^ | -0.204 | 0.581^**^ |
|  | (0.04) | (4.15) | (-0.45) | (3.06) |
| IADL | 0.509 | -0.324 | 0.0387 | -0.152 |
|  | (1.47) | (-0.99) | (0.13) | (-0.74) |
| Mobility | -0.148 | 1.328^***^ | -0.654^*^ | 1.014^***^ |
|  | (-0.63) | (7.82) | (-2.53) | (8.47) |
| Depression | -1.341^***^ | 1.601^***^ | -1.059^***^ | 1.382^***^ |
|  | (-5.28) | (8.37) | (-3.50) | (7.99) |
| Smoking | -0.283 | 0.283 | -0.354 | 0.193 |
|  | (-1.93) | (1.82) | (-1.67) | (1.15) |
| Overweight | -0.326^*^ | 0.191 | -0.468^**^ | -0.288^*^ |
|  | (-2.33) | (1.13) | (-3.09) | (-2.14) |
| Obese | -0.805^***^ | 0.697^***^ | -0.672^**^ | 0.351 |
|  | (-3.96) | (3.34) | (-2.94) | (1.95) |
| Heart disease#Female | 0.390 | 0.454 | -0.224 | 0.154 |
|  | (0.69) | (1.07) | (-0.47) | (0.65) |
| Hypertension#Female | -0.131 | 0.325 | -0.179 | -0.243 |
|  | (-0.66) | (1.61) | (-0.87) | (-1.43) |
| Stroke#Female | -0.866 | -0.788 | -0.0454 | 0.0576 |
|  | (-0.84) | (-1.16) | (-0.05) | (0.13) |
| Diabetes#Female | -0.276 | 0.132 | -0.420 | 0.163 |
|  | (-0.58) | (0.39) | (-1.00) | (0.68) |
| Lung Disease#Female | 1.180^*^ | -0.181 | -1.027 | 0.0133 |
|  | (2.24) | (-0.44) | (-1.79) | (0.04) |
| Cancer#Female | 0.778 | 0.204 | -0.376 | 0.0742 |
|  | (1.35) | (0.50) | (-0.81) | (0.25) |
| ADLA#Female | -0.161 | -0.278 | -0.349 | -0.479 |
|  | (-0.30) | (-0.83) | (-0.49) | (-1.94) |
| IADL#Female | -1.866^*^ | -0.516 | 0.0138 | 0.125 |
|  | (-2.10) | (-1.20) | (0.02) | (0.45) |
| Mobility#Female | -0.512 | -0.422^*^ | 0.0570 | 0.0570 |
|  | (-1.55) | (-2.08) | (0.17) | (0.38) |
| Depression#Female | 0.368 | 0.0445 | 0.482 | -0.0596 |
|  | (1.28) | (0.20) | (1.30) | (-0.28) |
| Smoking#Female | 0.260 | -0.0211 | 0.570 | -0.127 |
|  | (1.27) | (-0.09) | (1.82) | (-0.47) |
| Overweight#Female | -0.213 | -0.135 | 0.148 | 0.486^**^ |
|  | (-1.15) | (-0.61) | (0.69) | (2.59) |
| Obese#Female | -0.226 | -0.102 | -0.0920 | 0.219 |
|  | (-0.84) | (-0.38) | (-0.27) | (0.89) |
| Constant | 2.227^***^ | -2.898^***^ | 3.687^***^ | -4.903^***^ |
|  | (3.93) | (-4.35) | (4.12) | (-6.53) |
| var(u1) | 2.318^***^ | | 2.036^***^ | |
|  | (8.30) | | (6.30) | |
| var(u2) | 4.004^***^ | | 2.372^***^ | |
|  | (8.45) | | (8.21) | |
| Observations | 8404 | | 7597 | |
| *BIC* | 14918.2 | | 12685.4 | |

Note: t statistics in parentheses; ^*^ p < 0.05, ^**^ p < 0.01, ^***^ p < 0.001.

Table A1c: Regression results for Figure 2c, Italy

|  | 50-64 | | 65-79 | |
| --- | --- | --- | --- | --- |
|  | Very good | Poor | Very good | Poor |
| Female | -0.0744 | 0.0529 | -0.367^*^ | 0.0702 |
|  | (-0.54) | (0.31) | (-2.30) | (0.48) |
| Age | -0.0520^***^ | 0.0465^***^ | -0.0298^**^ | 0.0382^***^ |
|  | (-5.85) | (4.66) | (-2.97) | (4.59) |
| Native | -0.319 | -0.0105 | 0.158 | 0.600 |
|  | (-1.05) | (-0.03) | (0.39) | (1.74) |
| Below secondary edu. | 0.0884 | 0.494^***^ | 0.0136 | 0.485^***^ |
|  | (0.99) | (5.05) | (0.12) | (4.96) |
| Above secondary educ. | 0.512^***^ | -0.265 | 0.194 | -0.203 |
|  | (3.78) | (-1.52) | (1.04) | (-1.12) |
| Heart disease | -1.112^**^ | 1.714^***^ | -0.585^*^ | 1.129^***^ |
|  | (-3.17) | (7.14) | (-2.46) | (7.69) |
| Hypertension | -0.920^***^ | 0.382^**^ | -0.492^***^ | 0.348^***^ |
|  | (-6.86) | (2.65) | (-4.14) | (3.51) |
| Stroke | -0.941 | 1.576^**^ | -0.289 | 1.385^***^ |
|  | (-1.31) | (3.08) | (-0.50) | (4.58) |
| Diabetes | -1.135^***^ | 1.152^***^ | -0.877^***^ | 0.649^***^ |
|  | (-4.05) | (5.16) | (-4.37) | (4.84) |
| Lung disease | -1.755^**^ | 1.663^***^ | -1.047^**^ | 0.724^***^ |
|  | (-2.94) | (5.24) | (-2.84) | (3.94) |
| Cancer | -3.269^**^ | 2.670^***^ | -0.811 | 1.606^***^ |
|  | (-2.97) | (5.74) | (-1.44) | (5.70) |
| ADL | 0.254 | -0.158 | 0.586^**^ | 0.0915 |
|  | (1.08) | (-0.71) | (3.00) | (0.70) |
| IADL | 0.116 | -0.113 | -0.224 | -0.123 |
|  | (0.47) | (-0.48) | (-0.69) | (-0.87) |
| Mobility | -0.572^**^ | 1.111^***^ | -0.708^***^ | 0.736^***^ |
|  | (-3.20) | (8.82) | (-5.22) | (9.96) |
| Depression | -0.815^***^ | 1.097^***^ | -0.487^**^ | 0.957^***^ |
|  | (-4.58) | (7.16) | (-2.70) | (8.22) |
| Smoking | 0.105 | 0.00396 | -0.332 | 0.133 |
|  | (0.80) | (0.03) | (-1.88) | (0.88) |
| Underweight | -0.692 | -0.385 |  |  |
|  | (-0.52) | (-0.36) |  |  |
| Overweight | 0.0226 | -0.267 | -0.321^*^ | -0.189 |
|  | (0.18) | (-1.84) | (-2.51) | (-1.67) |
| Obese | -0.223 | -0.132 | -0.359 | -0.112 |
|  | (-1.19) | (-0.68) | (-1.87) | (-0.75) |
| Heart disease#Female | 0.750 | -0.832^*^ | 0.128 | -0.00733 |
|  | (1.36) | (-2.20) | (0.28) | (-0.03) |
| Hypertension#Female | 0.201 | 0.0428 | 0.0495 | 0.168 |
|  | (1.10) | (0.23) | (0.29) | (1.24) |
| Stroke#Female | 0.312 | -0.480 | -0.342 | 0.371 |
|  | (0.29) | (-0.68) | (-0.26) | (0.67) |
| Diabetes#Female | 0.0577 | -0.460 | 0.543 | -0.0141 |
|  | (0.14) | (-1.51) | (1.77) | (-0.07) |
| Lung Disease#Female | 0.0626 | -0.891^*^ | 0.891 | 0.337 |
|  | (0.08) | (-2.14) | (1.73) | (1.17) |
| Cancer#Female | 1.842 | -0.901 | 0.178 | -0.333 |
|  | (1.56) | (-1.69) | (0.25) | (-0.90) |
| ADLA#Female | 0.0914 | 0.391 | -0.197 | 0.00447 |
|  | (0.28) | (1.43) | (-0.64) | (0.03) |
| IADL#Female | -0.415 | -0.160 | -0.223 | 0.210 |
|  | (-1.11) | (-0.55) | (-0.51) | (1.08) |
| Mobility#Female | 0.00880 | -0.363^*^ | 0.0786 | -0.0353 |
|  | (0.04) | (-2.50) | (0.45) | (-0.38) |
| Depression#Female | 0.264 | 0.175 | -0.0116 | -0.0177 |
|  | (1.25) | (0.96) | (-0.05) | (-0.12) |
| Smoking#Female | -0.181 | 0.359 | 0.0665 | -0.353 |
|  | (-0.97) | (1.76) | (0.24) | (-1.61) |
| Underweight#Female | 0.441 | 1.474 |  |  |
|  | (0.32) | (1.30) |  |  |
| Overweight#Female | -0.170 | 0.536^**^ | 0.244 | 0.126 |
|  | (-1.02) | (2.87) | (1.33) | (0.83) |
| Obese#Female | -0.128 | 0.402 | 0.156 | 0.282 |
|  | (-0.52) | (1.61) | (0.57) | (1.37) |
| Constant | 3.213^***^ | -5.025^***^ | 1.681^*^ | -4.790^***^ |
|  | (5.39) | (-7.05) | (2.01) | (-6.75) |
| var(u1) | 2.050^***^ | | 1.221^***^ | |
|  | (8.77) | | (6.21) | |
| var(u2) | 2.202^***^ | | 1.333^***^ | |
|  | (8.26) | | (8.16) | |
| Observations | 8646 | | 8758 | |
| *BIC* | 16030.6 | | 14823.5 | |

Note: t statistics in parentheses; ^*^ p < 0.05, ^**^ p < 0.01, ^***^ p < 0.001.

Table A1d: Regression results for Figure 2d, Sweden

|  | 50-64 | | 65-79 | |
| --- | --- | --- | --- | --- |
|  | Very good | Poor | Very good | Poor |
| Female | 0.0161 | 0.315 | -0.0873 | -0.0227 |
|  | (0.09) | (1.36) | (-0.53) | (-0.13) |
| Age | -0.0362^**^ | 0.0535^***^ | -0.0702^***^ | 0.0302^**^ |
|  | (-3.08) | (3.99) | (-6.64) | (2.95) |
| Native | 0.788^***^ | -0.386^*^ | 0.709^***^ | -0.260 |
|  | (4.13) | (-2.15) | (3.74) | (-1.74) |
| Below secondary educ. | -0.436^***^ | 0.0613 | -0.412^***^ | 0.00444 |
|  | (-3.37) | (0.48) | (-3.49) | (0.04) |
| Above secondary educa. | 0.552^***^ | -0.116 | 0.453^***^ | -0.137 |
|  | (4.26) | (-0.82) | (3.51) | (-1.08) |
| Heart disease | -1.491^***^ | 0.829^***^ | -1.115^***^ | 0.370^**^ |
|  | (-4.91) | (3.52) | (-6.01) | (2.66) |
| Hypertension | -0.657^***^ | 0.162 | -0.738^***^ | -0.0468 |
|  | (-3.98) | (0.89) | (-5.47) | (-0.37) |
| Stroke | -0.900 | 0.560 | -1.012^**^ | 0.677^**^ |
|  | (-1.65) | (1.28) | (-3.06) | (3.03) |
| Diabetes | -1.454^***^ | 0.275 | -0.737^***^ | 0.693^***^ |
|  | (-5.00) | (1.15) | (-3.50) | (4.22) |
| Lung disease | -1.037^*^ | 0.835 | -1.318^**^ | 1.274^***^ |
|  | (-1.96) | (1.85) | (-3.23) | (4.94) |
| Cancer | -0.800^*^ | 0.967^*^ | -0.620^*^ | 0.936^***^ |
|  | (-2.09) | (2.51) | (-2.50) | (4.90) |
| ADL | -1.316^***^ | 0.862^**^ | -0.711^*^ | 0.385^*^ |
|  | (-3.51) | (3.29) | (-2.18) | (2.51) |
| IADL | -1.229^*^ | 0.197 | 0.153 | -0.0241 |
|  | (-2.07) | (0.49) | (0.52) | (-0.14) |
| Mobility | -0.810 | 0.628^**^ | -1.326^***^ | 0.605^***^ |
|  | (-1.79) | (3.01) | (-4.53) | (5.83) |
| Depression | -1.239^***^ | 1.232^***^ | -0.369 | 1.025^***^ |
|  | (-4.98) | (5.62) | (-1.59) | (5.93) |
| Smoking | -0.481^*^ | 0.203 | -0.160 | 0.155 |
|  | (-2.34) | (0.94) | (-0.74) | (0.73) |
| Underweight | 1.739 | 1.204 | 1.290 | 0.699 |
|  | (1.48) | (0.75) | (1.16) | (0.69) |
| Overweight | -0.430^**^ | 0.136 | -0.322^*^ | 0.0522 |
|  | (-2.61) | (0.67) | (-2.36) | (0.37) |
| Obese | -0.706^**^ | 0.689^**^ | -0.777^***^ | 0.407^*^ |
|  | (-2.94) | (2.76) | (-3.57) | (2.30) |
| Heart disease#Female | 0.235 | -0.442 | 0.0492 | 0.112 |
|  | (0.45) | (-1.28) | (0.16) | (0.52) |
| Hypertension#Female | -0.141 | 0.141 | -0.259 | 0.196 |
|  | (-0.61) | (0.59) | (-1.39) | (1.13) |
| Stroke#Female | -0.0837 | 0.185 | -0.0986 | 0.256 |
|  | (-0.09) | (0.31) | (-0.18) | (0.75) |
| Diabetes#Female | 0.178 | -0.0745 | -0.151 | 0.224 |
|  | (0.37) | (-0.21) | (-0.45) | (0.93) |
| Lung Disease#Female | -0.328 | -0.0435 | -0.229 | -0.441 |
|  | (-0.45) | (-0.08) | (-0.40) | (-1.29) |
| Cancer#Female | 0.107 | -0.928^*^ | -0.256 | -0.738^**^ |
|  | (0.22) | (-1.96) | (-0.75) | (-2.73) |
| ADLA#Female | 0.233 | -0.433 | 0.766^*^ | -0.0825 |
|  | (0.43) | (-1.39) | (2.01) | (-0.38) |
| IADL#Female | -0.379 | 0.364 | 0.173 | -0.00246 |
|  | (-0.42) | (0.69) | (0.48) | (-0.01) |
| Mobility#Female | -0.735 | 0.0560 | 0.601 | 0.173 |
|  | (-1.40) | (0.23) | (1.88) | (1.30) |
| Depression#Female | 0.105 | -0.0562 | -0.461 | -0.0547 |
|  | (0.36) | (-0.22) | (-1.64) | (-0.26) |
| Smoking#Female | 0.0208 | 0.0475 | 0.171 | 0.0524 |
|  | (0.08) | (0.18) | (0.57) | (0.19) |
| Underweight#Female | -1.560 | 0.418 | -2.069 | -0.859 |
|  | (-1.17) | (0.24) | (-1.70) | (-0.78) |
| Overweight#Female | -0.230 | -0.0869 | -0.0495 | -0.0553 |
|  | (-1.04) | (-0.34) | (-0.25) | (-0.29) |
| Obese#Female | -0.202 | -0.0315 | 0.0566 | -0.0543 |
|  | (-0.63) | (-0.10) | (0.19) | (-0.23) |
| Constant | 3.059^***^ | -5.046^***^ | 5.476^***^ | -3.655^***^ |
|  | (4.21) | (-5.88) | (6.98) | (-4.75) |
| var(u1) | 3.259^***^ | | 3.471^***^ | |
|  | (8.83) | | (10.33) | |
| var(u2) | 1.388^***^ | | 1.451^***^ | |
|  | (4.81) | | (6.73) | |
| Observations | 5967 | | 7935 | |
| *BIC* | 10200.6 | | 14354.5 | |

Note: t statistics in parentheses; ^*^ p < 0.05, ^**^ p < 0.01, ^***^ p < 0.001.

Table A2: Estimated gender- and age-specific means of depression scale CES-D and Euro-D by SRH controlling for physical conditions; RE-models

|  | Males | | Females |  |
| --- | --- | --- | --- | --- |
|  | 50-64 | 65-79 | 50-64 | 65-79 |
| *U.S.*: CES-D |  |  |  |  |
| Excellent | 1.1 [1.1; 1.1] | 0.9 [0.8; 0.9] | 1.3 [1.3; 1.3] | 1.2 [1.2; 1.2] |
| Very good | 1.1 [1.1; 1.1] | 0.9 [0.9; 0.9] | 1.4 [1.4; 1.4] | 1.3 [1.3; 1.3] |
| Good | 1.3 [1.3; 1.4] | 1.1 [1.1; 1.1] | 1.7 [1.7; 1.7] | 1.5 [1.5; 1.5] |
| Fair | 1.8 [1.8; 1.9] | 1.4 [1.4; 1.4] | 2.3 [2.3; 2.3] | 2.0 [2.0; 2.0] |
| Poor | 2.7 [2.7; 2.8] | 2.0 [2.0; 2.1] | 3.3 [3.2; 3.3] | 2.6 [2.6; 2.6] |
| *Germany*: Euro-D |  |  |  |  |
| Excellent | 1.1 [1.1; 1.2] | 1.3 [1.1; 1.4] | 1.7 [1.6; 1.8] | 1.7 [1.5; 1.9] |
| Very good | 1.2 [1.1; 1.3] | 1.1 [1.1; 1.2] | 1.8 [1.7; 1.8] | 1.7 [1.6; 1.8] |
| Good | 1.5 [1.4; 1.6] | 1.4 [1.3; 1.4] | 2.2 [2.1; 2.2] | 2.0 [1.9; 2.1] |
| Fair | 2.2 [2.1; 2.3] | 1.9 [1.9; 2.0] | 3.1 [3.1; 3.2] | 2.9 [2.8; 3.0] |
| Poor | 3.6 [3.4; 3.9] | 3.4 [3.2; 3.6] | 4.6 [4.4; 4.8] | 4.2 [4.0; 4.3] |
| *Italy*: Euro-D |  |  |  |  |
| Excellent | 1.4 [1.3; 1.5] | 1.5 [1.4; 1.6] | 1.9 [1.8; 2.1] | 2.2 [2.0; 2.4] |
| Very good | 1.4 [1.3; 1.5] | 1.6 [1.5; 1.8] | 2.1 [2.0; 2.2] | 2.2 [2.1; 2.4] |
| Good | 1.7 [1.7; 1.8] | 1.9 [1.8; 1.9] | 2.5 [2.4; 2.6] | 2.7 [2.6; 2.8] |
| Fair | 2.5 [2.4; 2.7] | 2.7 [2.6; 2.8] | 3.7 [3.6; 3.8] | 3.8 [3.7; 3.9] |
| Poor | 4.8 [4.4; 5.1] | 4.7 [4.5; 4.9] | 5.1 [4.9; 5.3] | 5.7 [5.5; 5.8] |
| *Sweden*: Euro-D |  |  |  |  |
| Excellent | 1.2 [1.1; 1.2] | 1.2 [1.1; 1.2] | 1.7 [1.7; 1.7] | 1.6 [1.5; 1.6] |
| Very good | 1.3 [1.2; 1.4] | 1.3 [1.2; 1.3] | 1.9 [1.8; 2.0] | 1.7 [1.7; 1.8] |
| Good | 1.5 [1.5; 1.6] | 1.4 [1.4; 1.5] | 2.2 [2.1; 2.3] | 2.0 [2.0; 2.1] |
| Fair | 2.0 [1.9; 2.2] | 1.9 [1.8; 2.0] | 3.1 [2.9; 3.2] | 2.7 [2.6; 2.9] |
| Poor | 3.6 [3.1; 4.0] | 3.3 [3.1; 3.6] | 4.1 [3.8; 4.4] | 4.0 [3.8; 4.3] |

Note: All models control for physical conditions listed in Table 1.
